# Is gas/air tamponade essential for eyes with small peripheral retinal breaks without detachment during vitrectomy?

**DOI:** 10.1186/s12886-022-02401-2

**Published:** 2022-04-22

**Authors:** Kyung Ho Lee, Yoo-Ri Chung, Suji Yeo, Kihwang Lee

**Affiliations:** Department of Ophthalmology, Ajou University School of Medicine, 164 World Cup-ro, Yeongtong-gu, Suwon, 16499 South Korea

**Keywords:** Laser photocoagulation, Retinal break, Tamponade, Vitrectomy

## Abstract

**Background:**

To investigate the safety of vitrectomy with laser photocoagulation in eyes with small peripheral retinal breaks without air or gas tamponade.

**Methods:**

Among patients who underwent vitrectomy for various retinal disorders, those with small peripheral retinal breaks treated by laser photocoagulation without air or gas tamponade were included in this study. Their medical records were assessed retrospectively, and we investigated the characteristics of small peripheral retinal breaks and the incidence of postoperative retinal detachment (RD).

**Results:**

Thirty-one eyes of 31 patients who presented with small peripheral retinal breaks requiring endolaser photocoagulation during vitrectomy were included in this analysis. There were two cases of iatrogenic retinal breaks that occurred during vitrectomy, while others were preexisting lesions, including retinal tears, atrophic retinal holes, and retinal holes with lattice degeneration. There were no cases of RD during the follow-up period of at least 6 months.

**Conclusions:**

Adequate laser treatment without gas or air tamponade may be sufficient during vitrectomy in cases with small peripheral retinal breaks without concurrent RD, along with complete removal of vitreoretinal traction.

**Supplementary Information:**

The online version contains supplementary material available at 10.1186/s12886-022-02401-2.

## Background

Retinal breaks are a full-thickness opening in the neurosensory retina; a hole usually due to atrophy of the retina and often overlaid by an operculum, or a horseshoe-shaped, round, or slit-like tear. Retinal break with vitreoretinal traction can induce rhegmatogenous retinal detachment (RD) if left untreated. This is why retinal surgeons try to relieve any vitreoretinal traction as much as possible during vitrectomy to prevent secondary RD. In addition to laser treatment and sufficient removal of vitreoretinal traction around retinal breaks, air or gas tamponades are widely used to treat rhegmatogenous RD [1, 2].

Air or gas tamponade can also be applied in cases of retinal breaks without concomitant RD to prevent postoperative RD if noted preoperatively or intraoperatively. As retinal breaks are one of the main factors for RD development, air or gas tamponade is often preferred by many retinal surgeons to secure adequate time to stabilize the laser scar around the retinal break so that postoperative RD, the most common vision-threatening complication after vitrectomy, can be avoided [3]. However, air or gas tamponade can be associated with potential complications, including glaucoma, cataract, and unexpected migration to the anterior chamber [4]. Furthermore, patients need more time for the recovery of vision, as air or gas has to be resolved.

Theoretically, postoperative RD may be prevented without air or gas tamponade if vitreoretinal traction is thoroughly released and adequate laser treatment is applied around the retinal breaks without concomitant RD. Therefore, we investigated the incidence of postoperative RD in eyes treated with vitrectomy and balanced salt solution (BSS) tamponade in the presence of retinal breaks without concomitant RD.

## Methods

This study was approved by the Institutional Review Board of Ajou University Hospital, Suwon, Korea (IRB No.: AJIRB-MED-MDB-21-162) and complied with the Declaration of Helsinki. Patients who underwent vitrectomy at the Ophthalmology Department of Ajou University Hospital between January 2018 and September 2020 were included in this study. The exclusion criteria were as follows: (1) those who underwent vitrectomy due to rhegmatogenous retinal detachment; (2) those who received air, gas, or silicone oil tamponade at the time of vitrectomy; and (3) those who were followed-up for less than 6 months after vitrectomy.

Vitrectomies were performed for various retinal disorders, and simultaneous cataract surgery was performed by the operating surgeon. Axial lengths were measured using an IOL Master 500 (Carl Zeiss Meditec AG, Jena, Germany). Vitrectomy was performed using the Constellation® 25G System (Alcon Laboratories, Fort Worth, TX) by one of the two retinal specialists (K.L. or Y-R.C.) under the noncontact wide-angle viewing system (RESIGHT 700®, Carl Zeiss Meditec AG, Oberkochen, Germany). Posterior vitreous detachment (PVD) was induced near the optic disk by aspiration using a vitrectomy cutter or extrusion needle if needed. Removal of vitreous body was done at core and then at periphery with vitrectomy cutter facilitating scleral indentation if necessary, and triamcinolone was injected in cases that needed better visualization of vitreous. The vitreous near retinal lesions was thoroughly trimmed. Endolaser photocoagulation was performed in peripheral retinal lesions, including iatrogenic or preexisting retinal breaks. An 8–0 Vicryl suture was placed at the sclerotomy site if required when the leakage of BSS was noted.

Demographic factors, such as age and sex and ocular findings, including ocular disease, for vitrectomy were obtained retrospectively from the medical records. Categorical variables are presented as numbers (percentages), while numeric variables are presented as mean ± standard deviation and/or range.

## Results

A total of 593 vitrectomized eyes without gas or air tamponade were initially identified during the study period. Among these eyes, 31 eyes of 31 patients presented with small peripheral retinal breaks requiring endolaser photocoagulation during vitrectomy were finally included in this analysis. The mean age of the patients was 64.9 ± 9.7 years (range: 48–82 years), with 44% of the patients being men. We included nine pseudophakic eyes and 22 phakic eyes, and combined vitrectomy and cataract surgery were performed in 19 of 22 (86.4%) phakic eyes. PVD was induced in approximately half of the included eyes (48.4%, 15 of 31 eyes), while posterior vitreous was already detached in remaining 16 eyes. There were no cases that fluid-air exchange was needed to dry the edge of the retinal breaks. Baseline characteristics and preoperative diagnoses of the included eyes are summarized in Table 1 (described in detail in Additional file 1). The representative cases are described below.

**Table 1:** Demographic and preoperative characteristics of patients

Characteristic	N (%)
Age, years (mean ± SD)	64.9 ± 9.7
Sex, male	14 (45.2%)
Systemic disorders	
Diabetes	7 (22.6%)
Hypertension	14 (45.2%)
Right eye	13 (44.9%)
Axial length, mm (mean ± SD)	24.0 ± 1.2
Pseudophakic eye	9 (29.0%)
Preoperative diagnosis for vitrectomy	
Epiretinal membrane	17 (54.8%)
Intraocular lens subluxation/dislocation	5 (16.1%)
Vitreous hemorrhage	3 (9.7%)
Dropped crystalline lens fragments	3 (9.7%)
Lens subluxation/dislocation	1 (3.2%)
Traumatic intraocular foreign body	1 (3.2%)
Vitreomacular traction	1 (3.2%)

Data are expressed as number (percentage) unless stated

*SD* standard deviation

### Case with epiretinal membrane

**Fig. 1:**
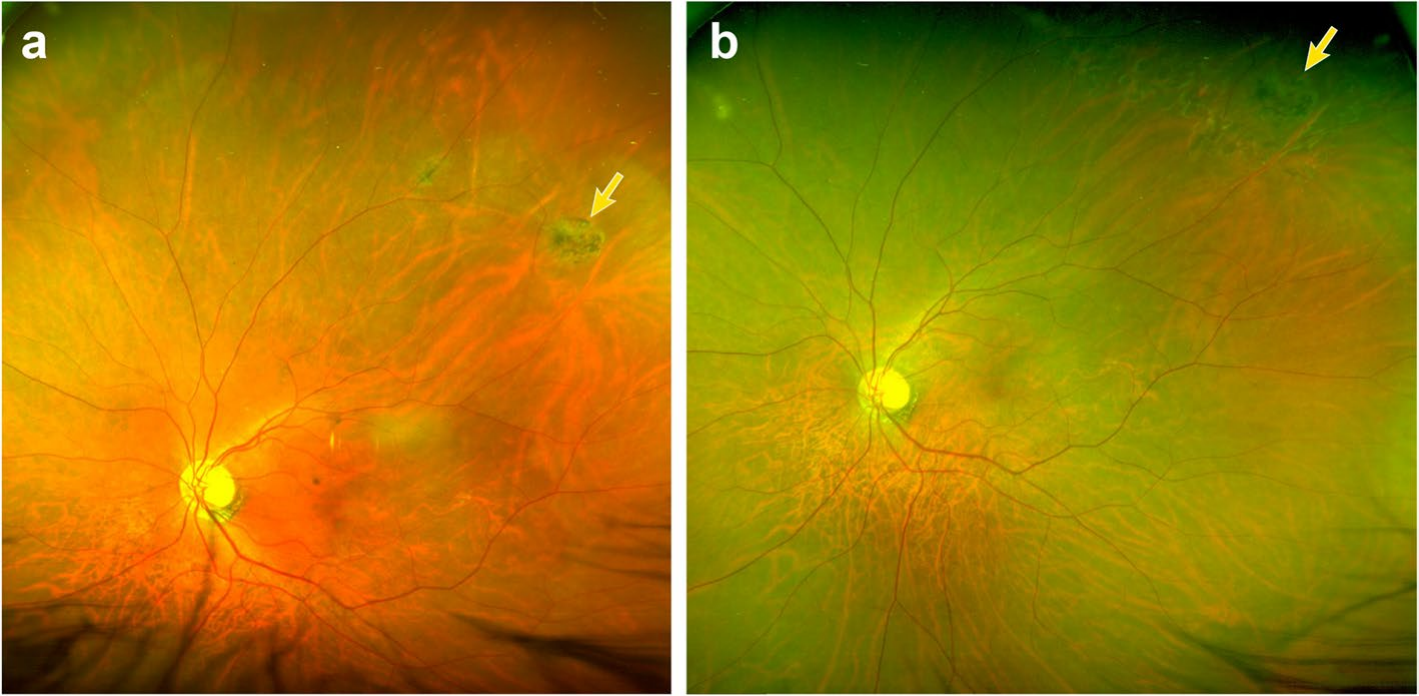
Preoperative (**A**) and postoperative after 12 months of vitrectomy (**B**) fundus photograph in a 62-years-old male patient who underwent vitrectomy for epiretinal membrane in the left eye. Lattice degeneration with an atrophic hole at 1 o’clock direction (*yellow arrows*) was noted preoperatively. Endolaser photocoagulation was applied for lattice degeneration, including the atrophic hole without gas or air tamponade, and the patient developed no retinal detachment for 12 months of follow-up

A 62-years-old man presented with dysmorphopsia in his left eye. The patient presented with an epiretinal membrane in his left eye; lattice degeneration with an atrophic hole in the superotemporal quadrant was noted preoperatively. The patient had undergone a vitrectomy in his right eye due to a full-thickness macular hole 3 years prior and had dyslipidemia as an underlying systemic disease. Vitrectomy, membranectomy, and peeling of the internal limiting membrane were performed to remove the epiretinal membrane. Endolaser photocoagulation was applied around the lattice degeneration, including the atrophic hole, and the surgery was completed. The patient did not develop RD after 12 months of follow-up, with stable laser scars around the retinal break (Fig. 1).

### Case with intraocular lens dislocation

**Fig. 2:**
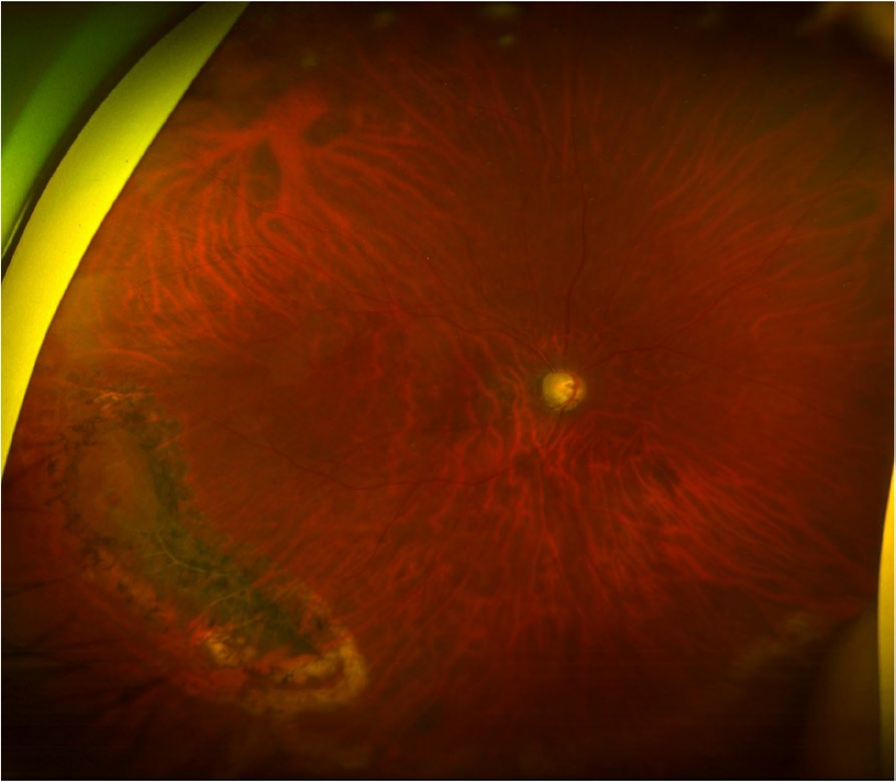
A postoperative fundus photograph was taken 12 months after vitrectomy for a dislocated intraocular lens in the right eye of a 49-years-old female patient. A retinal hole with lattice degeneration was noted at the inferotemporal quadrant. Similarly, endolaser photocoagulation was applied to the peripheral retinal lesion without gas or air tamponade, and the patient developed no retinal detachment for 24 months of follow-up

A 49-years-old woman presented with decreased vision in her right eye. The patient had previously undergone laser-assisted in situ keratomileusis 15 years ago and had undergone cataract surgery 5 years ago in both eyes. The patient had no systemic disorders and presented with a dislocated intraocular lens in her right eye, while lattice degeneration with a small retinal hole was noted at the inferotemporal periphery. Vitrectomy and exchange of intraocular lens with scleral fixation were performed, and endolaser photocoagulation was applied around the lattice degeneration, including an atrophic retinal hole. The patient did not develop RD for 24 months of follow-up, with stable laser scars around the lattice degeneration (Fig. 2).

### Cases with an iatrogenic retinal break during vitrectomy

Two patients who had prior panretinal photocoagulation due to proliferative diabetic retinopathy developed iatrogenic retinal breaks during vitrectomy. Both patients underwent vitrectomy for persistent vitreous hemorrhage. In one case (a 52-years-old man), an iatrogenic retinal tear occurred at the inferonasal mid-peripheral retina during PVD induction, while in the other case (a 56-years-old man), a retinal tear developed at the inferonasal mid-peripheral retina during removal of tractional fibrovascular proliferation. In both cases, endolaser photocoagulation was applied around the iatrogenic retinal break, and the surgery was completed. They did not develop RD at 25 and 14 months of follow-up, respectively.

Except for the cases mentioned above with iatrogenic retinal breaks, others had preexisting lesions, including retinal tears, retinal holes, and lattice degeneration (Table 2). Approximately half of the included eyes (16 of 31 eyes, 51.6%) presented with peripheral small retinal breaks at the superior retina. One patient had multiple peripheral lesions in the superonasal and inferonasal quadrants. There were no cases that developed RD during the mean follow-up period of 9.6 ± 5.2 months (range: 6–25 months). There were no cases that had or developed proliferative vitreoretinopathy during follow-up, and no patient required additional laser treatment.

**Table 2:** Type of surgeries and peripheral degenerations

Variables		N (%)
Type of surgery	Vitrectomy only	12 (38.7%)
	Combined surgery	19 (61.3%)
PVD induction during vitrectomy		15 (48.4%)
Location of retinal lesions	Inferonasal	7 (22.6%)
	Inferotemporal	8 (25.8%)
	Superonasal	8 (25.8%)
	Superotemporal	7 (22.6%)
	Multiple quadrants	1 (3.2%)
Types of retinal lesions	Iatrogenic break	2 (6.5%)
	Retinal tear	11 (35.5%)
	Retinal hole	5 (16.1%)
	Lattice degeneration	13 (41.9%)

*PVD* posterior vitreous detachment

## Discussion

Postoperative RD after vitrectomy is a complication that all retinal surgeons should avoid. The incidence of RD after vitrectomy is reported to be 1–16% [5], which has been reduced with the development of microincision vitrectomy surgery (MIVS). Iatrogenic retinal breaks related to the sclerotomy sites and vitrectomy itself were less noted in MIVS than 20-gauge conventional vitrectomy due to the higher cutting rate, slower fluidics, and the use of microcannula with the oblique and small size of sclerotomy [6–8]. However, studies are reporting no differences in the incidence rates of iatrogenic retinal breaks related to surgically induced PVD according to vitrectomy instruments [9]. In addition to iatrogenic retinal breaks, small peripheral retinal breaks without detachment, and other risk factors for postoperative RD, may also be found before or during vitrectomy.

Liquefied vitreous, vitreoretinal traction and the presence of retinal breaks are prerequisites for the development of rhegmatogenous RD [10]. Thus, the conventional approach to avoid postoperative RD is to relieve vitreoretinal traction and create a firm chorioretinal adhesion around vitreoretinal adhesive retinal breaks with gas or air tamponade. Extensive vitreous trimming near these breaks should be combined, as prominent residual anterior vitreous may cause anterior vitreoretinal traction and subsequent retinal breaks or RD postoperatively [11]. The main function of the gas or air tamponade is not to cover the retinal break but to reduce intraocular currents and prevent fluid entry through the breaks before chorioretinal adhesion is sufficiently strong [10]. Meanwhile, subretinal fluid can be removed by active transportation across retinal pigment epithelium (RPE) and also by passive hydrostatic and oncotic forces so that the retina remains attached [12, 13].

The laser treatment produces a coagulative effect that allows almost immediate adhesion between the neurosensory retina and RPE, and more quickly than cryotherapy [14]. Laser photocoagulation enhanced the adhesive force to 128% of normal levels within 24 h in an in vivo study [15]. In this study, laser treatment was applied intraoperatively during vitrectomy in every case; no cases developed RD postoperatively. Therefore, this study may reflect that the appropriate laser treatment and the mechanism that creates normal attachment of the retina to RPE around the margins of retinal break without detachment is sufficient to prevent postoperative RD theoretically, even in the absence of gas or air tamponade when vitreoretinal traction is removed completely.

Although intraocular gas or air usually resolves within several weeks, concerns exist for patients with phakic eyes due to cataract formation and those with scleral fixated intraocular lens due to possible intraocular lens displacement. Elevated ocular pressure and migration of gas or air into the anterior chamber are also possible complications. Moreover, visual recovery may be delayed until the intraocular gas or air is resolved. The clinical impact of this study is that BSS with adequate laser treatment is sufficient for patients at risk of potential complications associated with intraocular gas or air. It should be noted that cases with concurrent RD, which were excluded from this study, were treated with gas or silicone oil tamponade, implying the importance of these tamponades in the presence of RD.

The exact size of retinal breaks that do not require gas or air tamponade could not be defined in this study, while those within one disc diameter seemed to be safe without gas or air tamponade. Based on our study, the following cases might be safe to be considered as indications for not using tamponade: 1) single small sized retinal breaks regardless of the location, 2) adequate laser treatment around the lesion, 3) good trimming of the vitreous over the lesion and lattice degeneration, 4) well-induced PVD beyond the anterior border of the retinal break, and 5) removal of posterior hyaloid membrane. On the other hand, the cases with multiple holes, retinal tears with elevated edge, and the presence of proliferative vitreoretinopathy should be considered to be tamponed with gas, air or silicone oil.

This study has several limitations related to its retrospective nature and the small number of included patients. Further prospective studies may provide further evidence for treatment guidelines in vitrectomy with retinal breaks.

## Conclusions

In conclusion, thorough vitreous removal and adequate laser treatment may be sufficient during vitrectomy in cases with small peripheral retinal breaks without concurrent RD. In addition, this may provide clinical evidence to avoid unnecessary tamponade in selective cases receiving vitrectomy.

## Supplementary Information

**Additional file 1: Supplementary Table**. Ocular characteristics of included eyes.

## Data Availability

The datasets used and/or analyzed during the current study are available from the corresponding author on reasonable request.

## Abbreviations

BSS: Balanced salt solution
MIVS: Microincision vitrectomy surgery
PVD: Posterior vitreous detachment
RD: Retinal detachment
RPE: Retinal pigment epithelium
