# Supplementary material for: Is gas/air tamponade essential for eyes with small peripheral retinal breaks without detachment during vitrectomy?
Source: BMC Ophthalmol. 2022 Apr 22;22:186. doi: 10.1186/s12886-022-02401-2 (PMC9027820; doi:10.1186/s12886-022-02401-2)
Supplement: Supplementary file 1 — Additional file 1: Supplementary Table. Ocular characteristics of included eyes. [file 12886_2022_2401_MOESM1_ESM.pdf]

Supplementary Table. Ocular characteristics of included eyes

Case	Diagnosis for vitrectomy	Retinal break			Contralateral eye
		Type	Number	Location	
1	IOL dislocation	Hole	3	1 at SN, 1 at IN, and 1 at ST periphery	No specific finding
2	Epiretinal membrane	Hole / lattice	1	SN far periphery	Previous macular hole
3	Epiretinal membrane	Tear	1	SN far periphery	No specific finding
4	Epiretinal membrane	Lattice	1	IN far periphery	Epiretinal membrane, retinal tear
5	Epiretinal membrane	Lattice	1	IT far periphery	Drusen
6	Epiretinal membrane	Lattice	1	IT far periphery	No specific finding
7	Epiretinal membrane	Lattice	1	ST far periphery	No specific finding
8	Lens dislocation	Lattice	1	ST far periphery	No specific finding
9	Epiretinal membrane	Tear	1	IN far periphery	No specific finding
10	Epiretinal membrane	Hole	1	SN far periphery	No specific finding
11	Epiretinal membrane	Hole	1	IT far periphery	No specific finding
12	Epiretinal membrane	Tear	1	IN far periphery	No specific finding
13	Epiretinal membrane	Tear	1	IT far periphery	Epiretinal membrane
14	Vitreomacular traction	Tear	1	SN far periphery	No specific finding
15	Epiretinal membrane	Tear	1	ST far periphery	No specific finding
16	IOL dislocation	Hole	1	IT far periphery	No specific finding
17	Epiretinal membrane	Hole	1	IN far periphery	Drusen
18	Epiretinal membrane	Lattice	1	ST far periphery	No specific finding
19	Vitreous hemorrhage	Tear	1	IN mid-periphery	PDR, PRP done
20	Epiretinal membrane	Lattice	1	IN far periphery	Epiretinal membrane

21	Epiretinal membrane	Tear	1	ST far periphery	No specific finding
22	IOL dislocation	Lattice	1	SN equator	No specific finding
23	Epiretinal membrane	Lattice	1	SN far periphery	No specific finding
24	Intraocular foreign body	Tear	1	SN mid-periphery	No specific finding
25	IOL dislocation	Hole / lattice	1	IT far periphery	Lattice
26	Vitreous hemorrhage	Tear (iatrogenic)	1	IN mid-periphery	PDR, PRP done
27	IOL dislocation	Lattice	1	IT far periphery	No specific finding
28	Lens subluxation	Tear	1	ST far periphery	No specific finding
29	Vitreous hemorrhage	Tear (iatrogenic)	1	IN mid-periphery	PDR, PRP done
30	Lens drop	Lattice	1	SN far periphery	No specific finding
31	Lens drop	Tear	1	ST far periphery	No specific finding

IN: inferonasal, IOL: intraocular lens, IT: inferotemporal, PDR: proliferative diabetic retinopathy, PRP: panretinal photocoagulation, SN: superonasal, ST: superotemporal
